# Serving the King's Men

**Published:** 1918-10-05

**Authors:** 


### Serving the King's Men.

MR. F. A. MCKENZIE, the well-known war correspondent, knows how to write a good appeal. It runs a little to length, but it would be difficult to describe in a few words the work of the Salvation Army with our fighting men in France or elsewhere. To anyone who has a boy serving in the war, whether he is a son or otherwise related (and there must be few who have not), an account of how those who most need mothering are mothered must be irresistible, and to them especially the little book issued by the Salvation Army under the above title can be commended. Countless mothers and fathers must be unspeakably grateful to this great body, to the Church Army, the Young Men's Christian Association, and other organisations for kindness of a very practical kind shown to their sons. This is work which is helping to win the war, for to keep up the hearts and attend to the comforts of our fighting men is work of supreme importance. The appeal is accompanied by a set of accounts, excellently arranged, of the income and expenditure of the War Fund. In the year ending September 30, 1917, half a million of money was received and spent; approximately two-thirds of this sum is represented by the turnover of food, etc., sold to sailors, soldiers, and munition workers at cost price.

